# Phosphoglucomutase comes into the spotlight

**DOI:** 10.1093/jxb/erac513

**Published:** 2023-03-13

**Authors:** Sofía Doello, Karl Forchhammer

**Affiliations:** Interfaculty Institute of Microbiology and Infection Medicine, University of Tübingen, Germany; Interfaculty Institute of Microbiology and Infection Medicine, University of Tübingen, Germany

**Keywords:** Cyanobacteria, glycogen, phosphoglucomutase, starch, *Synechocystis*

## Abstract

This article comments on:

**Ortega-Martínez P, Roldán M, Díaz-Troya S, Florencio FJ**. 2023. Stress response requires an efficient glycogen and central carbon metabolism connection by phosphoglucomutases in cyanobacteria. Journal of Experimental Botany **74**, 1532–1550

**Phosphoglucomutase (PGM) has recently gained attention as a key regulatory point in the metabolism of carbon storage compounds. In this issue, Ortega-Martínez *et al*. (2023) advance our understanding of the contribution of the different PGM isoforms to the carbon flux between glycogen and the central carbon metabolism in the cyanobacterium *Synechocystis.***

Glycogen is a rapidly metabolizable storage form of glucose that acts as an essential metabolic buffer in many organisms. It consists of β-1,4-linked d-glycosyl units that are interconnected through β-1,6 branching points, forming a large, hydrosoluble polymer. With its large accessible surface-to-volume ratio, it allows for rapid storage and mobilization of glucose units (Colpaert *et al*., 2020). Glycogen is widely distributed in prokaryotes and appears to be present in all cyanobacteria, which use it as energy storage for night-time survival (Gründel *et al*., 2012). Several cyanobacterial strains additionally contain forms of a semi-crystalline polyglucan, related to starch, the crystalline glucose storage polymer of plants (Ball *et al*., 2011). Phylogenetic reconstruction suggests that during plant evolution, primordial glycogen metabolism in the common ancestor of Archaeplastida was modified into starch metabolism through modifications of branching/debranching reactions (Ball *et al*., 2011; Chabi *et al*., 2021). Besides its role in survival during dark periods, glycogen metabolism has been proved to be essential in the model cyanobacterial strain *Synechocystis* sp. PCC 6803 for acclimation to macronutrient deficiency, in particular nitrogen (Gründel *et al*., 2012; Hickman *et al*., 2013), and long-term survival in a chlorotic state (Klotz *et al*., 2016). In addition to serving as a form of carbon storage, glycogen metabolism plays an important role in balancing energy homeostasis, allowing the control of the intracellular energy charge to prevent the over-reduction of electron carriers and collapse of the electron transport chain (Cano *et al*., 2018).

Glycogen and starch metabolism require the initial synthesis of glucose-1-phosphate (G1P) from glucose-6-phosphate (G6P), a reaction catalysed by the bi-directional enzyme phosphoglucomutase (PGM). This reaction represents a pivotal point connecting central carbon metabolism with glycogen synthesis/degradation (Stiers *et al*., 2017). Despite this central position in the metabolic network, cyanobacterial PGMs had received little attention until recently, perhaps because the synthesis of ADP-glucose from G1P by ADP-glucose pyrophosphorylase (AGP) was considered the rate-limiting step in glycogen formation (Ballicora *et al*., 2003) (see Box 1).

**Box 1. Schematic representation of the glycogen biosynthetic and catabolic pathway**

Phosphoglucomutases catalyse the interconversion of glucose-1-phosphate (G1P) and glucose-6-phosphate (G6P), a reaction that connects synthesis and degradation of glycogen to the central carbon metabolism. During glycogen synthesis, G1P is obtained from G6P via the phosphoglucomutase reaction and converted to ADP-glucose (ADP-Glc) by the ADP-glucose pyrophosphorylase (AGP) for subsequent polymerization by the glycogen synthase (GlgA) and branching enzymes (GlgB). Glycogen degradation is mediated by the glycogen phosphorylase (GlgP) and debranching enzymes (GlgX) to yield G1P, which is then converted to G6P by the phosphoglucomutases. In *Synechocystis*, two enzymes are capable of carrying out the phosphoglucomutase reaction: Sll0726 (PGM), which is responsible for 99% of the phosphoglucomutase activity, and Slr1334 (PGM/PMM). Although Slr1334 only has a minor contribution in the interconversion of G1P and G6P, it is able to catalyse an additional reaction of great importance: the synthesis of glucose-1,6-bisphosphate (G1,6BP) from G1P or G6P and fructose-1,6-bisphosphate (F1,6BP) (represented in blue). G1,6BP functions as an activator compound for Sll0726, thus regulating a fundamental reaction for carbon metabolism.

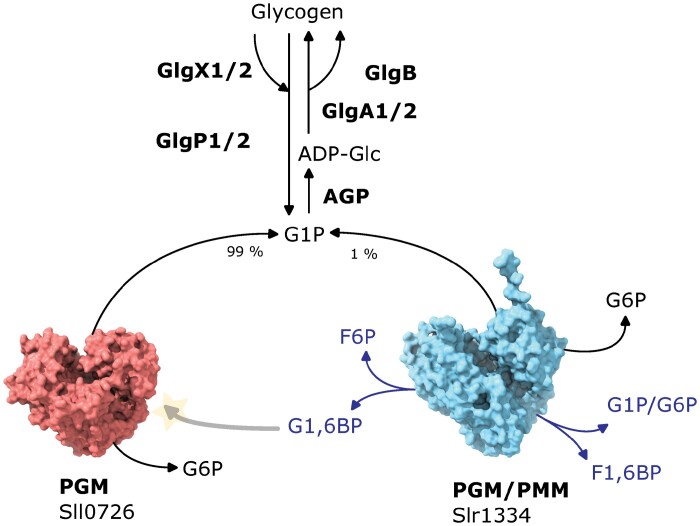

For most of the enzymes involved in glycogen synthesis and degradation, *Synechocystis* harbours two genes encoding two different isoforms that are required under different physiological conditions (Yoo *et al*., 2014; Doello *et al*., 2018; Koch *et al*., 2019; Neumann *et al*., 2022b, Preprint). PGM is no exception to this: the genes corresponding to the Cyanobase ORFs *sll0726* and *slr1334* encode potential phosphoglucomutases. The *sll0726* product (PGM) is highly homologous to other bacterial phosphoglucomutases and was suggested to represent a target of thioredoxin regulation (Lindahl and Florencio, 2003), while the enzyme encoded by *slr1334* was predicted to encode a phosphogluco/phosphomannomutase bifunctional enzyme (PGM/PMM) (Liu *et al*., 2013). Until recently, only a single published study addressed the question of the differential function of the two potential phosphoglucomutases by a genetic approach (Liu *et al*., 2013). Whereas PGM (Sll0726), which was shown to provide most of the phosphoglucomutase activity, could be inactivated, segregation of PGM/PMM (Slr1334) knockout failed. This suggested an essential function of this gene, although PGM/PMM contributed only a very minor part of the total phosphoglucomutase activity in cell extracts. Further awareness of the importance of glycogen metabolism was raised by studies of the acclimation to nitrogen starvation in *Synechocystis* (Doello *et al*., 2018; Neumann *et al*., 2021), regulation of diurnal metabolism (Selim *et al*., 2021), or its connection to polyhydroxybutyrate (PHB) formation (Koch *et al*., 2019). Recent biochemical analyses gave further insights into the function and regulation of PGM (Doello *et al*., 2022; Neumann *et al*., 2022a, see below).

The present work by Ortega-Martínez *et al*. (2023), in this issue of JXB, represents an important step towards a deeper understanding of cyanobacterial glycogen metabolism by revisiting the functional significance of phosphoglucomutase for the overall physiology of *Synechocystis*. The authors revealed that PGM is 10 times more abundant than PGM/PMM, and confirmed by mutant analysis that PGM contributes 99% of the total PGM activity. To find out how limitation of carbon flux to and from glycogen affects *Synechocystis* physiology under various environmental perturbations, the phenotype of PGM, PGM/PMM, and AGP mutants was systematically analysed. Although the PGM mutant could still produce a residual amount of glycogen, it showed similar growth defects to an AGP-deficient strain, which is completely devoid of glycogen. The study confirmed the requirement of glycogen metabolism for efficient acclimation to periods of nitrogen starvation, and for night-time survival with long night phases (16 h). In contrast, no defect was observed when cells were grown with short night periods (8 h). Glycogen synthesis and PGM activity were also found to be required for acclimation to high light conditions as the PGM- and AGP-deficient mutants lost viability upon prolonged exposure to a photosynthetic photon flux density of 200 μmol photons s^–1^ m^–2^. However, PGM and AGP mutants showed different responses towards high salt acclimation. Whereas the PGM-deficient strain could tolerate 0.5 M NaCl treatment, the AGP-deficient mutant was unable to do so, probably because of its inability to synthesize the compatible solute glucosylglycerol (GG), which needs ADP-glucose. Strikingly, almost wild-type levels of GG were detected in the PGM-deficient strain, despite the very low residual PGM activity ascribable to the presence of PGM/PMM. Overexpression of PGM/PMM in a PGM-deficient background using a strong promoter increased the total phosphoglucomutase activity to ~20% of wild-type levels. This was sufficient to phenotypically compensate for the lack of PGM, as all growth and acclimation defects of the PGM-deficient mutant could be complemented. This confirmed that PGM/PMM can in fact carry out phosphoglucomutase activity. Measuring the cellular levels of G1P and G6P yielded a remarkable result: although glycogen synthesis requires conversion of G6P to G1P and blocking this pathway should result in increased levels of G6P, the PGM mutant showed higher G1P levels under all tested conditions as compared with the wild type, whereas G6P only accumulated during nitrogen deprivation. Overexpression of PGM/PMM reverted the metabolite concentrations to the wild-type levels, confirming that the increased levels of G1P and G6P in the PGM mutant were caused by the low phosphoglucomutase activity of PGM/PMM. To clarify whether PGM/PMM is an essential protein in *Synechocystis*, Ortega-Martínez *et al*. expressed an additional copy of the *slr1334* gene under a tuneable P_*arsB*_ promoter, which allowed deletion of the respective wild-type locus. As long as the P_*arsB*_ promoter was turned on by the addition of arsenite, cells could grow. However, in the absence of the inducer, the cells stopped growing, clearly demonstrating that *slr1334* is an essential gene in *Synechocystis*.

Altogether, this study opens up intriguing new questions regarding the role of glycogen metabolism in stress acclimation. This work coincides with and perfectly complements two recent studies on the function of PGM in *Synechocystis*. Doello *et al*. (2022) revealed a novel regulatory mechanism to control PGM activity. During nitrogen starvation, the enzyme is phosphorylated on a conserved regulatory seryl residue near the catalytic site, which inhibits its activity. This preserves the glycogen stores by preventing carbon flux from glycogen into central metabolism during prolonged periods of nitrogen starvation. Upon resuscitation, when combined nitrogen is available again, the enzyme is dephosphorylated, restoring its activity and allowing efficient glycogen mobilization upon demand. Intriguingly, a conserved phosphorylation event has been reported for the mammalian PGM homologue, suggesting that these regulatory mechanisms may be widely distributed. A second study revealed the molecular function of the product of *slr1334*, which was annotated as PGM/PMM. Neumann *et al*. (2022a) revealed that Slr1334 catalyses the synthesis of the activator molecule for phosphoglucomutase, glucose-1,6-bisphosphate (G1,6BP), using G1P or G6P and fructose-1,6-bisphosphate (F1,6BP) as substrates (see Box 1). How this activator compound is synthesized in bacteria was so far unknown. Slr1334 belongs to a distinct family of phosphohexomutases, which is present in many cyanobacteria, deep branching bacteria, and archaea, and it appears that members of this family represent prokaryotic G1,6BP synthases. This function may provide a straight-forward explanation for the puzzling physiological role of Slr1334. Mutants that are deficient in glycogen metabolism are perfectly viable under non-stressing growth conditions, whereas depletion of Slr1334 abrogates cell growth, as shown by Ortega-Martínez *et al*. (2023). This strongly indicates a role for Slr1334 beyond glycogen metabolism. The activator molecule G1,6BP might be necessary for the activation of vital functions, as it has been reported that G1,6BP can activate other enzymes, such as the essential phosphoglucosamine mutase in *Escherichia coli* (Jolly *et al*., 1999).

These recent studies place the phosphoglucomutase reaction into the spotlight for a better understanding of cyanobacterial glycogen metabolism. To emerge is the picture of a highly sophisticated network of metabolic regulation in which a key role is played by the phosphorylated and bisphopshorylated sugar molecules. Our knowledge on how glycogen turnover provides robustness to the maintenance of cellular homeostasis is fundamental to metabolic engineering of cyanobacteria with the aim of using them as green cell factories.
